# A comparison between the therapeutic effects of Conbercept combined with panretinal photocoagulation and panretinal photocoagulation monotherapy for high-risk proliferative diabetic retinopathy

**DOI:** 10.3389/fendo.2022.1038757

**Published:** 2023-01-13

**Authors:** Yaoyao Sun, Huijun Qi

**Affiliations:** ^1^ Department of Ophthalmology, Peking University People’s Hospital, Beijing, China; ^2^ Eye Diseases and Optometry Institute, Beijing, China; ^3^ Beijing Key Laboratory of Diagnosis and Therapy of Retinal and Choroid Diseases, Beijing, China; ^4^ College of Optometry, Health Science Centre, Peking University, Beijing, China

**Keywords:** Conbercept, panretinal photocoagulation, high-risk PDR, anti-VEGF (vascular endothelial growth factor), neovascularization

## Abstract

**Objective:**

To compare the therapeutic effects of the administration of intravitreal Conbercept (IVC) plus panretinal photocoagulation (PRP) to that of PRP monotherapy in patients with high-risk proliferative diabetic retinopathy (PDR).

**Methods:**

In this retrospective consecutive case series, we analyzed the data on high-risk PDR patients followed up for 12 months. Patients were divided into two groups: the IVC+PRP group and the PRP monotherapy group. Patients in the IVC+PRP group were initially administered 3 IVC injections and PRP, while patients in the PRP monotherapy group received PRP only. Depending on the grouping criteria, patients in both groups were administered either IVC+PRP or PRP only if the neovascularization (NV) did not regress. From the initiation to month 12 of treatment, we recorded and compared the data on the NV regression rate, improvement in best-corrected visual acuity (BCVA), laser spots, changes in central macular thickness (CMT), complications, and the need for vitrectomy for all patients.

**Results:**

In this study, 79 eyes of 58 patients in the IVC+PRP group and 86 eyes of 60 patients in the PRP monotherapy group were included. During the follow-up of 12 months, the number of eyes with complete regression, partial regression, and no regression or increase in NV were 56 (70.88%), 23 (29.12%), and 0 (0%) in the IVC+PRP group and 13 (15.12%), 50 (58.14%), and 23 (26.74%) in the PRP group (*p* < 0.001). The BCVA was significantly higher and CMT was lower in the patients of the IVC+PRP group than in the PRP monotherapy group at 3, 6, and 12 months of follow-up (*p* < 0.05). The mean number of laser spots was lower in the patients of the IVC+PRP group than in the PRP group (1,453 ± 87 spots vs. 2,267 ± 94 spots, *p* < 0.05). A significantly lower percentage of patients in the IVC+PRP group underwent vitrectomy than that in the PRP group (7 (8.86%) vs. 27 (31.40%), *p* < 0.001).

**Conclusion:**

High-risk PDR patients treated with IVC + PRP showed a higher rate of NV regression, more effective improvement in the BCVA, and lower vitrectomy rate compared to those who were administered PRP monotherapy.

## Introduction

Diabetic retinopathy (DR) is the main retinal complication of diabetes mellitus (DM) and the leading cause of loss of vision and blindness in working-age people (1–3). Proliferative diabetic retinopathy (PDR) is characterized by neovascularization (NV) of the optic disc or vitreous and pre-retinal hemorrhage, which finally develops into a tractional retinal detachment. A study found that the average percentage of PDR in all DM patients was 6.96% (6.87–7.04), suggesting that around 17 million PDR patients worldwide are at risk of losing their eyesight (2).

High-risk PDR occurs when NV is accompanied by vitreous hemorrhage or when NV of the disc (NVD) occupies more than or equal to one-quarter to one-third of the disc area, even in the absence of vitreous hemorrhage, indicating severe ischemia (4). Bressler et al. found that high-risk PDR had a higher probability of advancing PDR, e.g. more vitrectomies of vitreous hemorrhage or tractional retinal detachment were needed for patients with high-risk PDR than that required for patients with moderate PDR, even after intensive treatment, such as retinal photocoagulation (5).

Panretinal photocoagulation (PRP) has been used as a classical treatment for PDR for over 40 years. In this procedure, the ischemic regions of the peripheral retina are eliminated to decrease NV while maintaining central vision. PRP also significantly lowers the probability of severe loss of vision in patients with high-risk PDR by inducing retinal NV regression. In high-risk PDR individuals, PRP should be administered at the earliest to effectively reduce retinal NV and PDR progression (4, 6, 7). Anti-vascular endothelial growth factor (anti-VEGF) agents, including ranibizumab and aflibercept, can facilitate the regression of NV while eliminating diabetic macular edema (DME), and hence, are recommended for treating high-risk PDR patients (8, 9). By investigating different PDR treatment modalities, the RELATION study showed that PDR patients with DME benefited more from Ranibizumab+PRP combined therapy than from PRP monotherapy. The PROTEUS study found that Ranibizumab+PRP therapy was more effective than PRP monotherapy in preventing the recurrence of NV with fewer PRP treatment times over 12 months (10, 11).

Conbercept is a member of the recombinant VEGF decoy receptor class. It is a recombinant fusion protein consisting of the constant region and the third and fourth Ig domains of VEGFR2, as well as, the second Ig domain of VEGFR1 (12, 13). Intravitreal administration of Conbercept (IVC) is effective in treating PDR and DME cases. Treatment with IVC combined with PRP has a greater effect on functional outcomes than PRP monotherapy, including improvements in the visual acuity of the patients and reduction of macular edema (14). However, as studies on the therapeutic effects of IVC+PRP on high-risk PDR patients are limited, further research on this treatment method for high-risk PDR should be encouraged. We conducted a retrospective consecutive case series study to compare the therapeutic effects of the combined treatment using IVC plus PRP to those of PRP monotherapy in high-risk PDR patients.

## Methods

### Study design

This study had a retrospective consecutive case series design. Following the guidelines of the Declaration of Helsinki, informed consent forms were signed by all participants after they received information on the risks of IVC and PRP therapy. The study was approved by the Medical Ethics Committee of Peking University People’s Hospital.

### Patients

In total, 118 high-risk PDR patients (165 eyes) who visited the Department of Ophthalmology, Peking University People’s Hospital, from September 2016 to April 2021, were recruited in this study. All patients underwent a follow-up of 12 months. The patients were placed either in the IVC+PRP group (79 eyes) or the PRP monotherapy group (86 eyes). The inclusion criteria were as follows: 1) Patients primarily diagnosed with high-risk PDR and confirmed by color fundus photography (CFP) and/or fluorescein angiography (FA) (CFP and FA both conducted using the Optos PLC 200TX, Dunfermline; United Kingdom); 2) Those who were followed up for at least 12 months; 3) Patients who underwent IVC+PRP combined therapy or PRP monotherapy. The exclusion criteria were as follows: 1) Patients with other retinal disorders like rhegmatogenous retinal detachment, uveitis, epiretinal membrane, age-related macular degeneration, high myopia fundus changes, and ocular tumors; 2) Patients who were administered intraocular treatment other than IVC and PRP, such as intravitreal injections of other anti-VEGF agents or steroid components or macular grid pattern photocoagulation; 3) Patients who underwent any intraocular surgery within 6 months before participation; 4) Patients with a proliferative membrane because of PDR. The clinical data of the patients at 3, 6, 9, and 12 months were recorded and compared. The data collected 15 days before or after 3, 6, and 9 months and 30 days before or after 12 months were considered to be the data corresponding to 3, 6, 9, and 12 months, respectively.

### Treatment

Panretinal photocoagulation (PRP) was conducted according to a previously described protocol (Lumenis Novus Omni, Lumenis Be, Inc. San Jose, USA) (15). A level II to level III reaction for retinal photocoagulation was identified; the exposure time and the spot size were 0.3 s and 300 µm, respectively. The photocoagulation scope was two papilla diameters (PD) away from the temporal side of the macula and from both the upper and lower vascular arcades on the retina to the peripheral retina, and 1 PD away from the nasal side of the optic disc to the peripheral retina. Conbercept (0.05 mL/0.5 mg; Chengdu Kanghong, China) was administered to all patients of the IVC+PRP group. Intravitreal injections were performed according to a previously reported method (16). In the IVC+PRP group, the initial treatment included the administration of three IVC injections, once every four weeks. PRP was performed simultaneously, following the diagnosis of high-risk PDR, and was completed within two weeks. The patients in the PRP monotherapy group, however, received PRP treatment only. Three months after the start of treatment, fundus fluorescein angiography (FFA) was performed in both groups. Patients in the PRP monotherapy group underwent re-treatment with photocoagulation if the NV did not regress. Similarly, for the IVC+PRP group, if NV persisted, IVC+PRP was administered again, regardless of the presence of DME.

### Efficacy and safety assessments

The general and medical information of the patients was recorded at the beginning before eye treatment was started. The data on the age, gender, body mass index, blood pressure, and fasting glucose level of the patients were recorded. All treated patients received standard ophthalmological examinations and optical coherence tomography (OCT) (Zeiss Cirrus HD-OCT5000, Carl Zeiss Meditec AG; Jena, Germany) during every visit. On the first visit and months 3, 6, 9, and 12, the best-corrected visual acuity (BCVA) of both eyes was checked and recorded using the Early Treatment Diabetic Retinopathy Study [ETDRS] letters. Visual acuity improvement of ≥2 lines was considered to be improved vision, while a decrease in visual acuity by ≥2 lines was considered to be a deterioration of visual acuity. The rest was considered to be unchanged visual acuity. Compared to the status of NV at baseline, the complete absence of NV was considered to be complete NV regression. Persistent or increased NV was considered to be the absence of NV regression or increase in NV. NV regression partially was considered to be “partial NV regression”.

Additionally, the intraocular pressure was evaluated at the first visit, as well as on months 3, 6, 9, and 12. Spectral domain-optical coherence tomography (SD-OCT) was performed on both eyes using an acquisition methodology for determining the macular thickness. Central macular thickness (CMT) was determined by SD-OCT examinations and was calculated as the combined thickness of the subretinal fluid and neurosensory retina. CMT increased ≥50 µm was considered to be increased CMT, while a decrease in CMT by ≥50 µm was considered to be decreased CMT. CMT change within 50 µm was considered an unchanged CMT. CFP was performed on all patients at each visit. FA was also performed at the first visit, as well as, after 3, 6, 9, and 12 months if the patient had no history of allergies and had normal hepatic and renal functions. From the beginning of treatment through month 12, the data on parameters, such as the NV regression status, improvement in BCVA, laser spots, changes in CMT, other complications, and the need for vitrectomy, for all patients were investigated and compared. The primary efficacy analysis was the NV regression rate. The number of eyes with complete regression, partial regression, no regression, or increased NV was divided by the number of total eyes treated and was calculated as as the NV regression rate. Other results were investigated as a secondary efficacy analysis. We also recorded systemic and ocular complications.

### Statistical analysis

The data were analyzed using the SPSS software (version 12.0). The Shapiro-WiIk test was conducted to check whether the data were normally distributed. Qualitative data were either analyzed by Chi-squared tests or Fisher’s exact tests. Quantitative data that were normally distributed were tested by independent samples t-tests, whereas non-normally distributed data were tested by Mann-Whitney U tests. All differences were considered to be statistically significant at *p* < 0.05.

## Results

### Baseline information

From September 2016 to April 2021, data on 165 eyes (118 patients) were recorded. Among all participants, 71 (60.17%) were men, and 47 (39.83%) were women; the mean age of all participants was 57.09 years, respectively. The baseline information is shown in **Table 1**. The differences in age, gender, body mass index, blood pressure, fasting glucose, BCVA, IOP, CMT, and area of NV between the patients in the IVC+PRP and PRP groups were not statistically significant (*p* > 0.05; **Table 1**).

**Table 1:** Demographic information for the two groups.

Group	IVC+PRP	PRP	p-value
Female, frequency (%)	24 (40.00)	23 (39.66)	0.56
Age, (mean ± SD), y	54.67 (13.3)	59.59 (16.9)	0.13
BMI, (mean ± SD), kg/m^2^	27.87 (2.2)	29.01 (2.7)	0.44
Systolic blood pressure, (mean ± SD), mmHg	134.62 (11.9)	138.11 (16.0)	0.68
Diastolic blood pressure, (mean ± SD), mmHg	78.84 (8.3)	77.65 (9.2)	0.52
fasting glucose, mmol/L	7.61 (2.3)	7.07 (2.8)	0.28
IOP, (mean ± SD), mmHg	16.7 (3.0)	16.9 (2.4)	0.54
NV area (mean ± SD) Disc Area (DA)	2.57 (1.4)	2.87 (1.6)	0.35
BCVA, (mean ± SD)	54.25 (21.6)	51.95 (25.5)	0.76
CMT, (mean ± SD), µm	325.05 (106.93)	302.90 (100.90)	0.51
No. of eyes with DME (CMT ≥250um)	35 (44.30%)	41 (47.67%)	0.66

### NV regression

**Figure 1:**
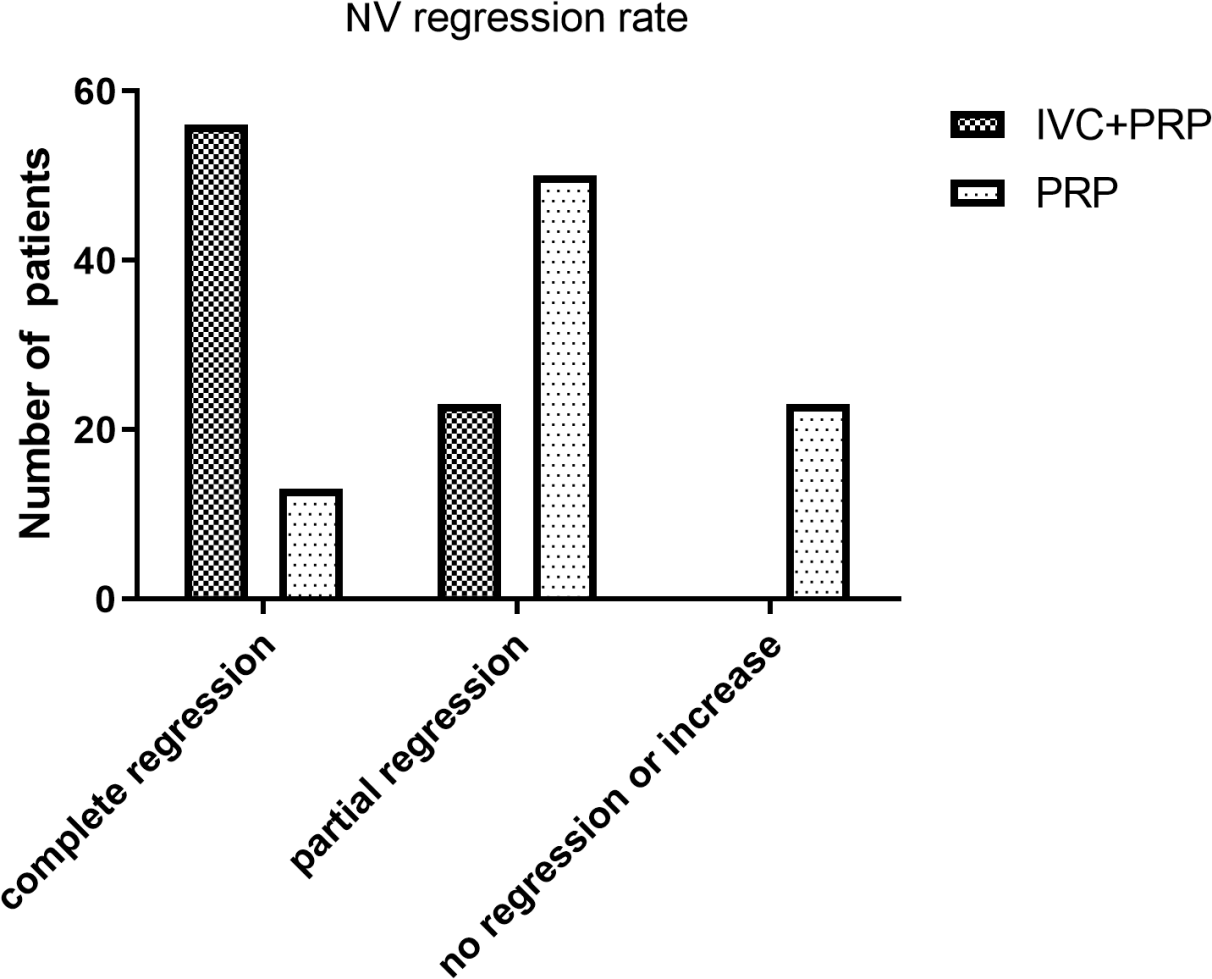
The NV regression rates of patients in both groups. Treatment with IVC combined with PRP showed a higher rate of NV regression at month 12.

The number of eyes with complete NV regression, partial regression, and no regression or increase was 56 (70.88%), 23 (29.12%), and 0 (0%), respectively, in the IVC+PRP group and 13 (15.12%), 50 (58.14%), and 23 (26.74%), respectively, in the PRP monotherapy group after 12 months of treatment compared to their corresponding values at baseline. The NV regression rate in the IVC+PRP group was significantly higher than that in the monotherapy group (*p* < 0.001; **Figure 1**).

### Changes in the BCVA

**Figure 2:**
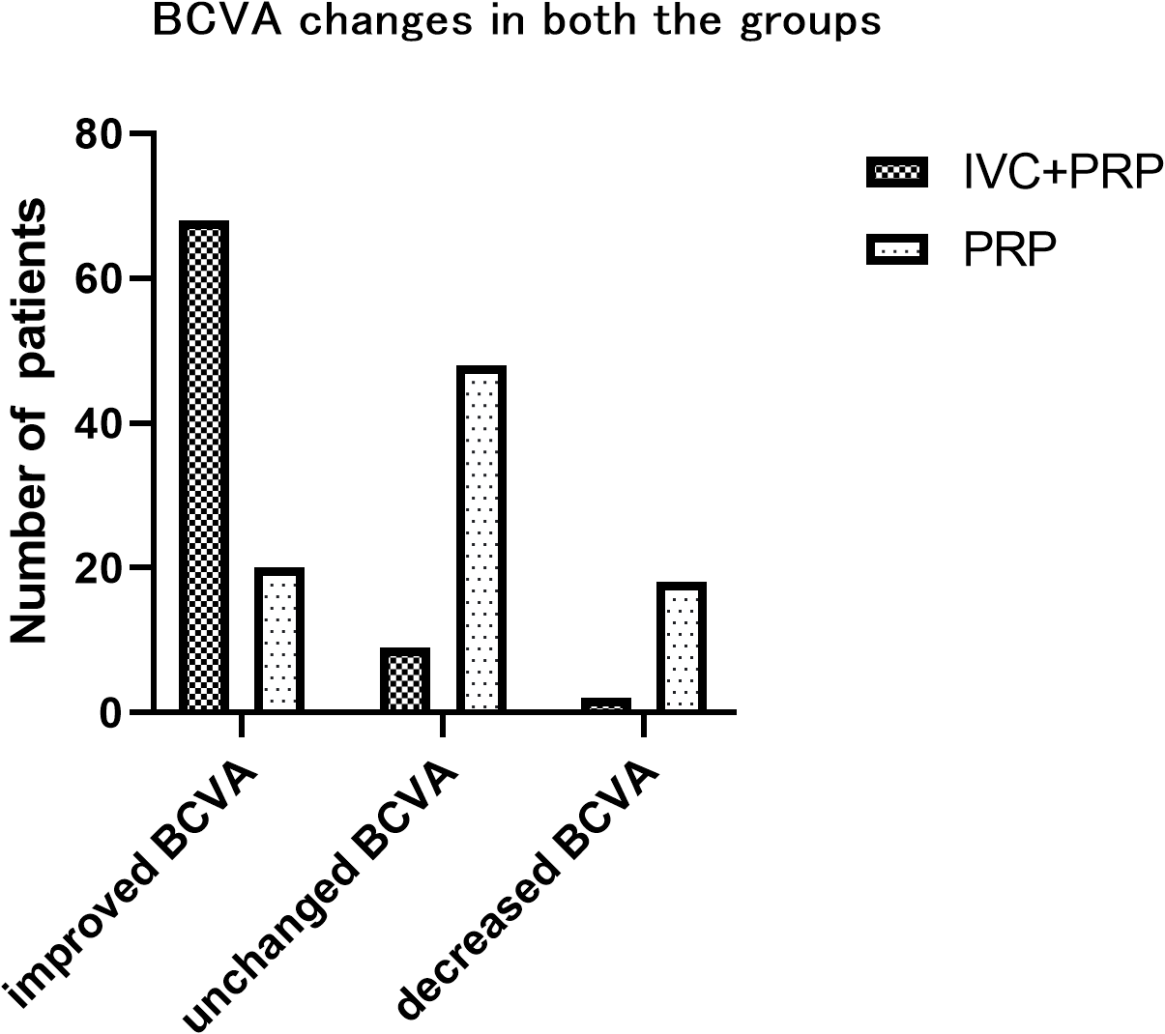
The BCVA changes in the patients of both groups. After 12 months, a significant difference in the BCVA changes was found between the groups for the number of eyes with improved BCVA, unchanged BCVA, and decreased BCVA.

**Figure 3:**
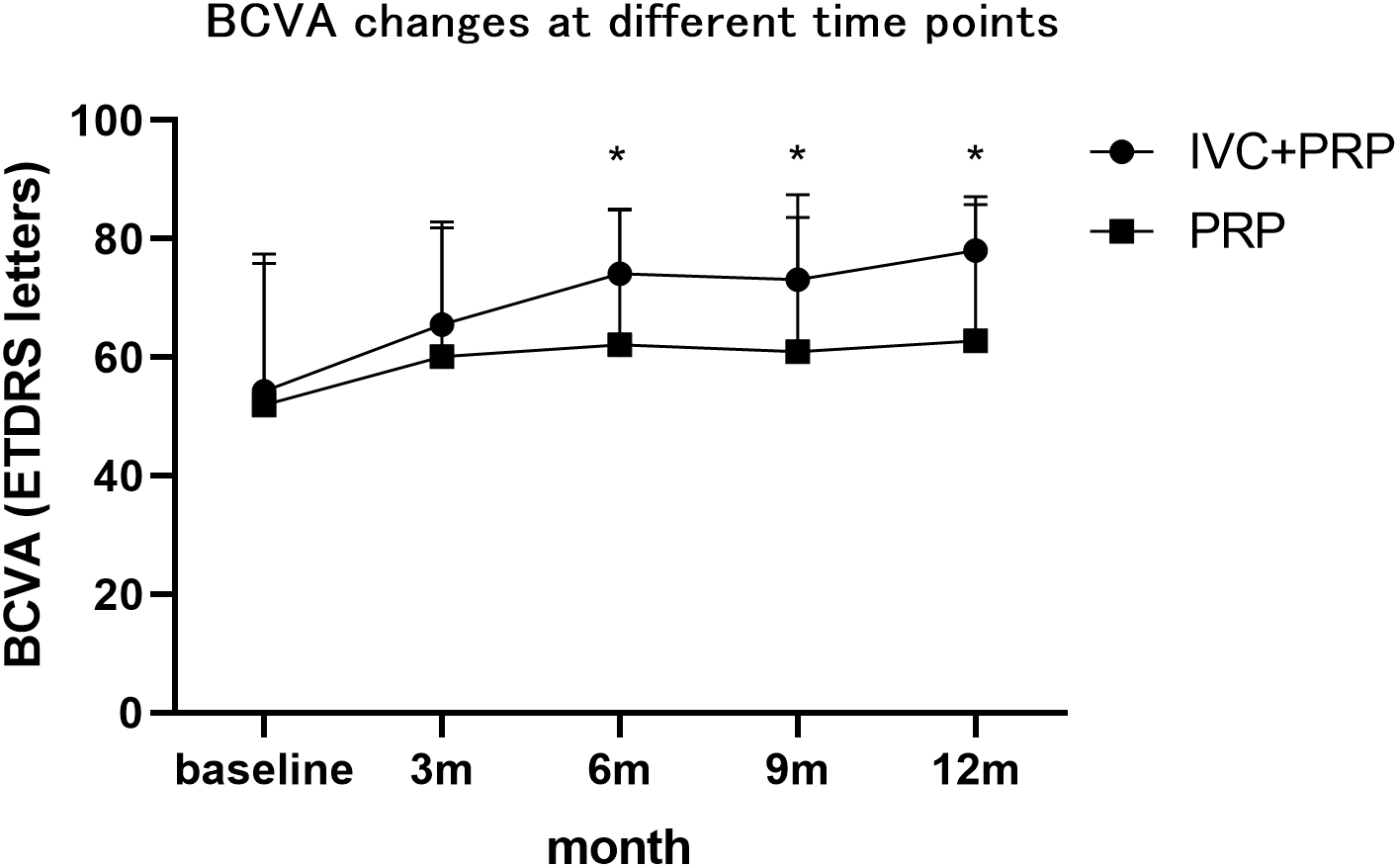
The BVCA changes at different time points. The average BCVA was greater in the patients of the IVC+PRP group than in those of the PRP monotherapy group at each visit, while significant differences were found at months 6, 9, and 12. *Demonstrates statistically significant difference.

At 12 months of follow-up, the number of eyes with improved, unchanged, and decreased BCVA was 68 (86.08%), 9 (11.39%), and 2 (2.53%), respectively, in the IVC+PRP group and 20 (23.26%), 48 (55.81%), and 18 (20.93%), respectively, in the PRP monotherapy group. The differences in the changes in the BCVA between the IVC+PRP and PRP groups were significant (*p* < 0.001; **Figure 2**). The average BCVA was significantly greater in the IVC+PRP group than in the PRP monotherapy group at each visit. Additionally, the differences were significant at months 6 (*p* = 0.042), 9 (*p* = 0.049), and 12 (*p* = 0.011; **Figure 3**).

### Changes in the CMT

**Figure 4:**
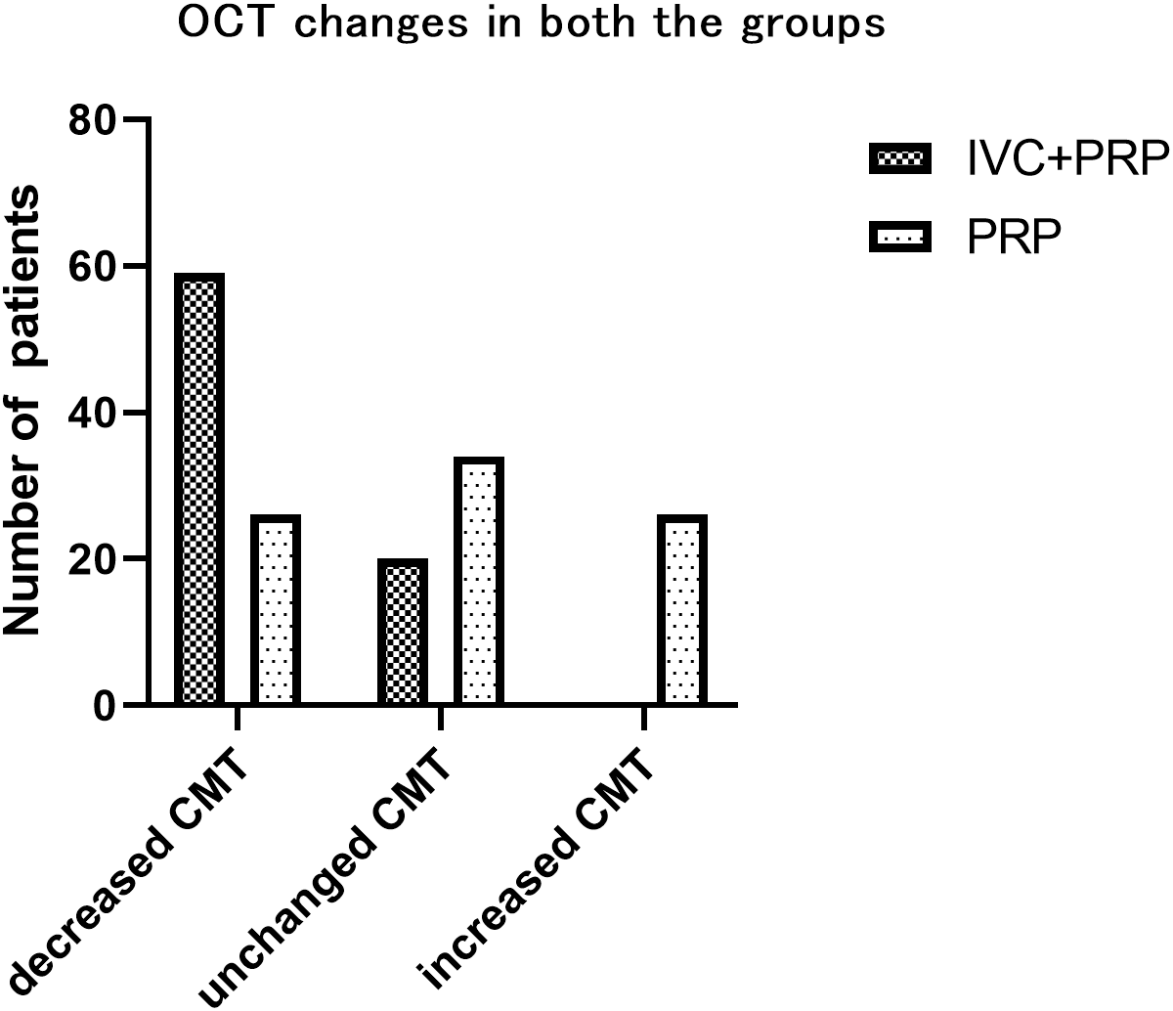
The changes based on OCT examinations in the patients of both groups. At month 12, a significant difference was found between the groups for the number of eyes with decreased CMT, unchanged CMT, and greater CMT.

**Figure 5:**
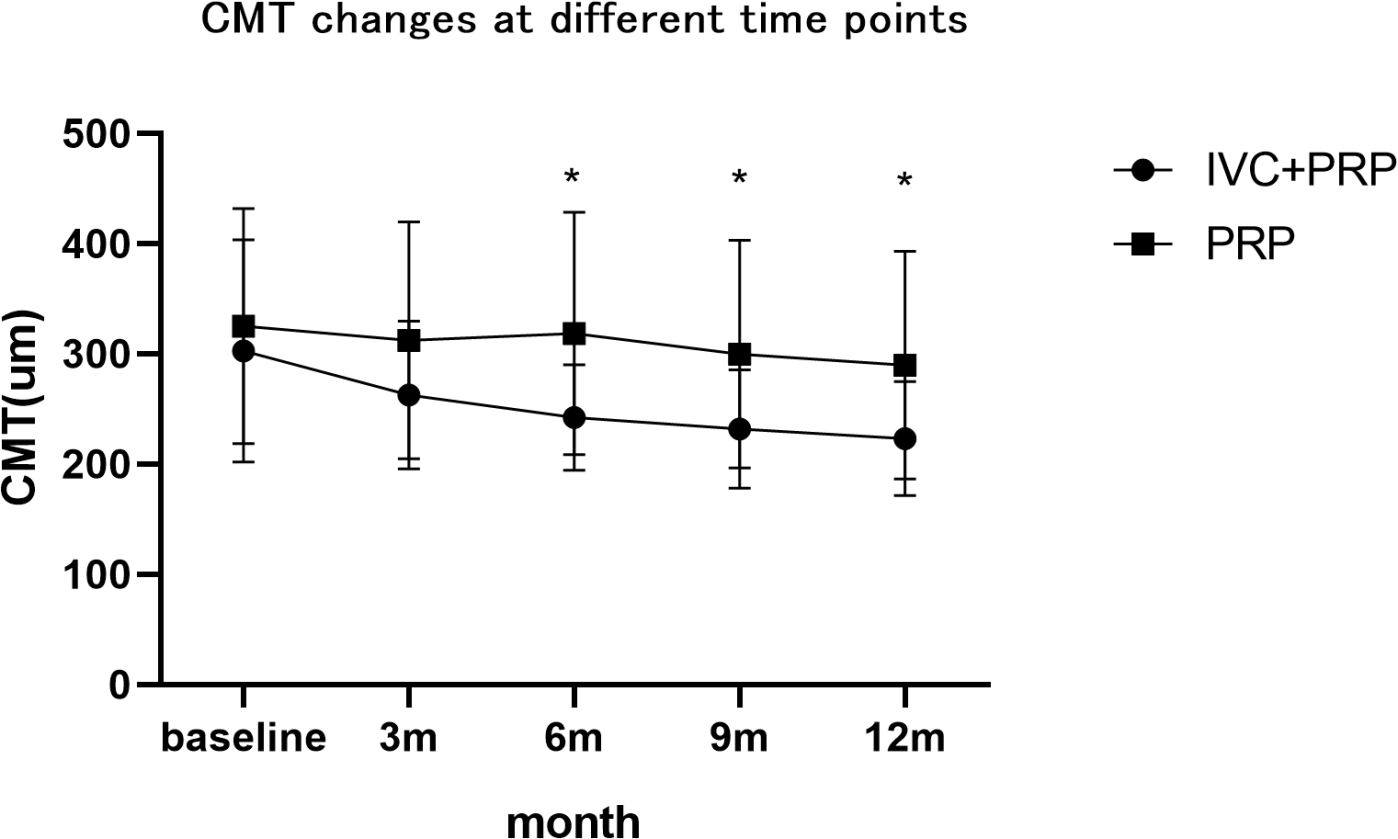
The changes in the CMT at different time points. The average CMT was lower in the patients of the IVC+PRP group than in those of the PRP monotherapy group during each visit, while significant differences were found at months 6, 9, and 12. *Demonstrates statistically significant difference.

After 12 months of treatment, the numbers of eyes with decreased, unchanged, and increased CMT were 59 (74.68%), 20 (25.32%), and 0(0%), respectively, in the IVC+PRP group and 26 (30.23%), 34 (39.54%) and 26 (30.23%), respectively, in the PRP monotherapy group. Significant differences were observed in the CMT between the groups (*p* < 0.001; **Figure 4**). The average CMT was significantly lower in the IVC+PRP group than in the PRP monotherapy group at each visit. Additionally, significant differences were recorded at months 6 (*p* = 0.07), 9 (*p* = 0.015), and 12 (*p* = 0.014; **Figure 5**).

### Other outcomes

**Figure 6:**
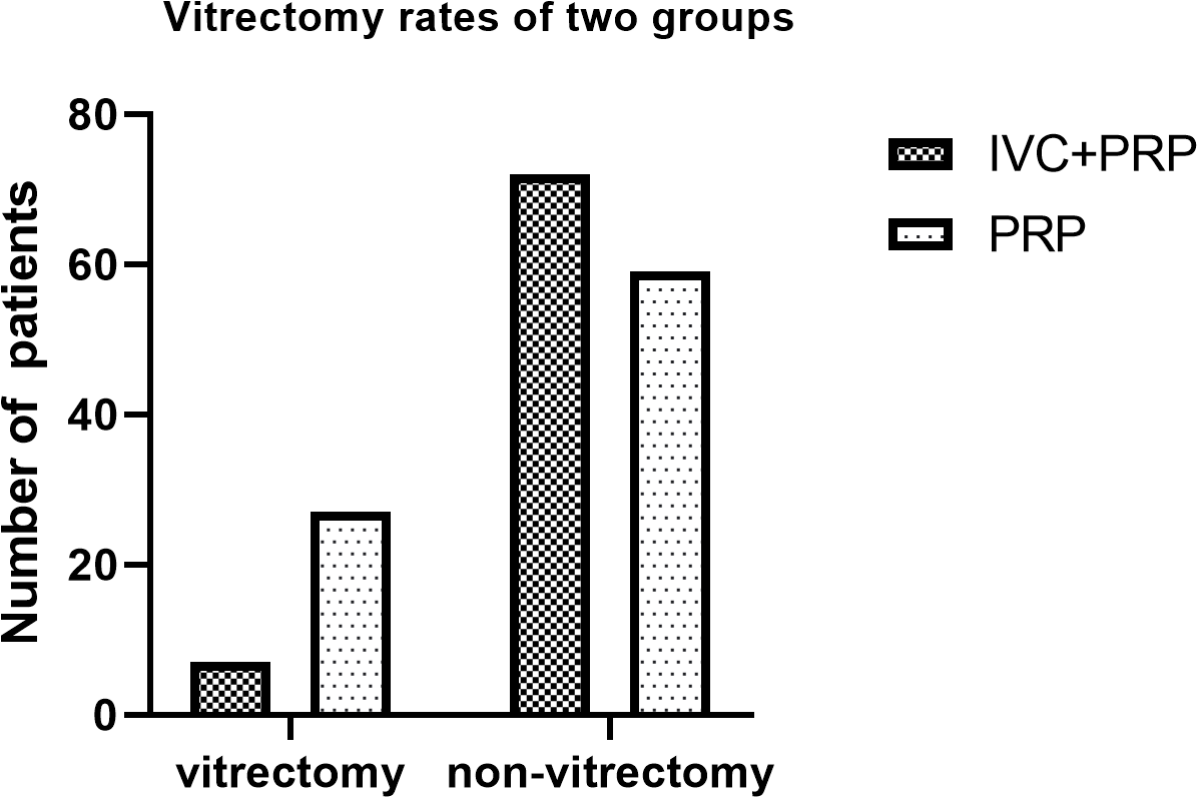
Vitrectomy rates of the patients in the two groups. The percentage of patients undergoing vitrectomy was significantly different between the groups.

The mean number of laser spots was significantly lower in the IVC+PRP group than in the PRP group (1,453 ± 87 spots vs. 2,267 ± 94 spots, *p* < 0.05). The difference in the total number of laser treatments between the groups was not significant. Patients in the IVC group received 4.95 ± 0.90 injections. In the IVC+PRP group, vitrectomy due to disease progression to severe vitreous hemorrhage was conducted on 7 eyes (8.86%). In the PRP monotherapy group, vitrectomy was conducted on 27 eyes (31.40%). The percentage of patients who required vitrectomy was statistically different between the groups (*p* < 0.001; **Figure 6**). Four eyes in the PRP group developed neovascular glaucoma, while no case of neovascular glaucoma was reported in the IVC+PRP group. No endophthalmitis, retinal tear, or cataract exacerbation due to the treatment or serious systemic side events were reported in either group.

## Discussion

Our results suggested that the treatment with IVC+PRP was more effective than PRP monotherapy in causing NV regression among high-risk PDR patients during a follow-up of 12 months. The effectiveness of an anti-VEGF agent combined with PRP in high-risk PDR was consistent with previously reported results. This was the first study to investigate the effects of combined treatment with IVC and PRP in high-risk PDR cases (10, 17).

The findings of our study were similar to those of previous studies, which suggested that PRP and anti-VEGF combination therapy can achieve optimal efficacy in treating high-risk PDR patients by enhancing BCVA and NV regression while decreasing the risk of adverse effects (18). Although PRP is a standard therapeutic strategy for PDR, in some studies, it was effective in only 60% of PDR patients, and the remaining 40% of the patients either underwent surgery or developed poor vision (19, 20). Some studies have shown that an increase in VEGF expression in PDR is closely related to hypoxia and inflammatory responses (21, 22). By phosphorylating tight-junction proteins, VEGF increases capillary permeability, which causes macular edema and angiogenesis. Thus, VEGF inhibition is necessary for anti-vascularization therapy in PDR patients. Anti-VEGF medication might be administered to prevent NV in high-risk PDR cases before the completion of PRP within the effective action period of the drug. This can prevent disease progression before the patients receive PRP treatment. PRP takes over three weeks from operation to display its full effects, whereas anti-VEGF therapy acts fast. Thus, it can be administered to avoid disease progression to the point of requiring vitreous surgery before the effects of PRP treatment are expressed. In our study, a lower percentage of patients in the combined treatment group underwent vitrectomy, which was similar to the results reported in other studies (23, 24). Anti-VEGF rescue therapy can also be used to manage some cases of PRP-treated PDR patients with persistent NVs, even in cases where neovascular regression cannot be achieved (25). Matteo et al. found insufficient information to compare PRP treatment and combined treatment using anti-VEGF and PRP for NV regression in a meta-analysis due to high inconsistencies among the included studies. However, after adjustments by surface under the cumulative ranking curve (SUCRA) analysis in this meta-analysis, the combination treatment was recommended (26).

The results of the other tested parameters showed that patients in the IVC+PRP group had better vision outcomes with lower CMT values. In patients with combined macular edema, vision improved mainly due to the remission of macular edema. In patients without macular edema, visual acuity improved due to the absorption of pre-retinal or inter-retinal hemorrhage caused by the regression of neovascularization. Anti-VEGF plays an important role in macular edema treatment. VEGF is the most significant molecule that needs to be broken down in the retinal barrier. Pathologically, hyperglycemia, protein kinase C activation, and advanced glycation end-product protein synthesis during DR and DME affect the production of VEGF. VEGF inhibitors are used to prevent inner blood-retinal barrier disruption and control DME (27–29). The decrease in retinal edema also facilitated the implementation of PRP and its early effects. Fewer laser spots were observed in the IVC+PRP group in our study, which was consistent with the PROTEUS study (10). Although Bressler et al. had concerns regarding the long-term benefits of anti-VEGF in PDR, the differences in the loss of vision between the anti-VEGF and PRP groups vanished after five years of follow-up. However, fewer laser treatments were required to reduce retinal damage and patient pain.

In this study, Conbercept, a newly developed therapeutic agent in China, was used as an anti-VEGF agent. Its treatment effects are mostly attributable to the VEGF family of factors (VEGF-A, B, C, and PIGF) that prevent the growth of NV and the reduction of vascular permeability in the retina (30). Xia et al. found that Conbercept can strongly inhibit inflammation, angiogenesis, and oxidative response in the PDR model by reducing macrophage inflammatory protein-1 (MIP-1), intercellular cell adhesion molecule-1 (ICAM-1), IL-1β, IL-6, and TNF-α protein levels (31). Concerning the improvement of vision, a meta-analysis showed that Conbercept with PRP greatly increased the overall effectiveness and decreased the central thickness of the macula and other complications compared to the condition of the patients in the control group (14). Previous studies concentrated more on improving visual acuity and reducing macular edema in patients. We found that treatment with IVC+PRP was more effective than treatment with PRP in facilitating the regression of NV in PDR patients. Thus, the administration of Conbercept should be continued in the clinical setting.

All VEGF inhibitors have relatively short half-lives, while PRP treatment has a permanent effect. Thus, PRP is the preferred and major method to treat PDR (32). Our study showed that PRP monotherapy caused the regression of NV in 73.26% of eyes (total and partial regression). The Diabetic Retinopathy Study showed that PRP significantly lowered the risk of severe visual loss in patients with high-risk PDR. PRP is regarded as the gold standard for treating PDR cases (8, 19) and is recommended as the first-line treatment for PDR when anti-VEGF therapy is not available due to difficulties in frequent follow-ups or financial reasons (33).

The main limitation of this study was that this was a single-center, retrospective study with a follow-up time of only 12 months. Thus, prospective, randomized, and multicenter studies with a longer follow-up are needed to comprehensively compare the effects of IVC+PRP treatment to those of PRP monotherapy in PDR. Also, as the study was a retrospective one, we could not obtain more information on various aspects of the patients, including visual changes, non-perfusion areas, etc.

To summarize, treatment with IVC combined with PRP caused a higher rate of NV regression, greater improvement in the BCVA, and also decreased the need to perform vitrectomy in patients with high-risk PDR, compared to monotherapy with PRP.

### Typical cases

#### Case 1

**Figure 7:**
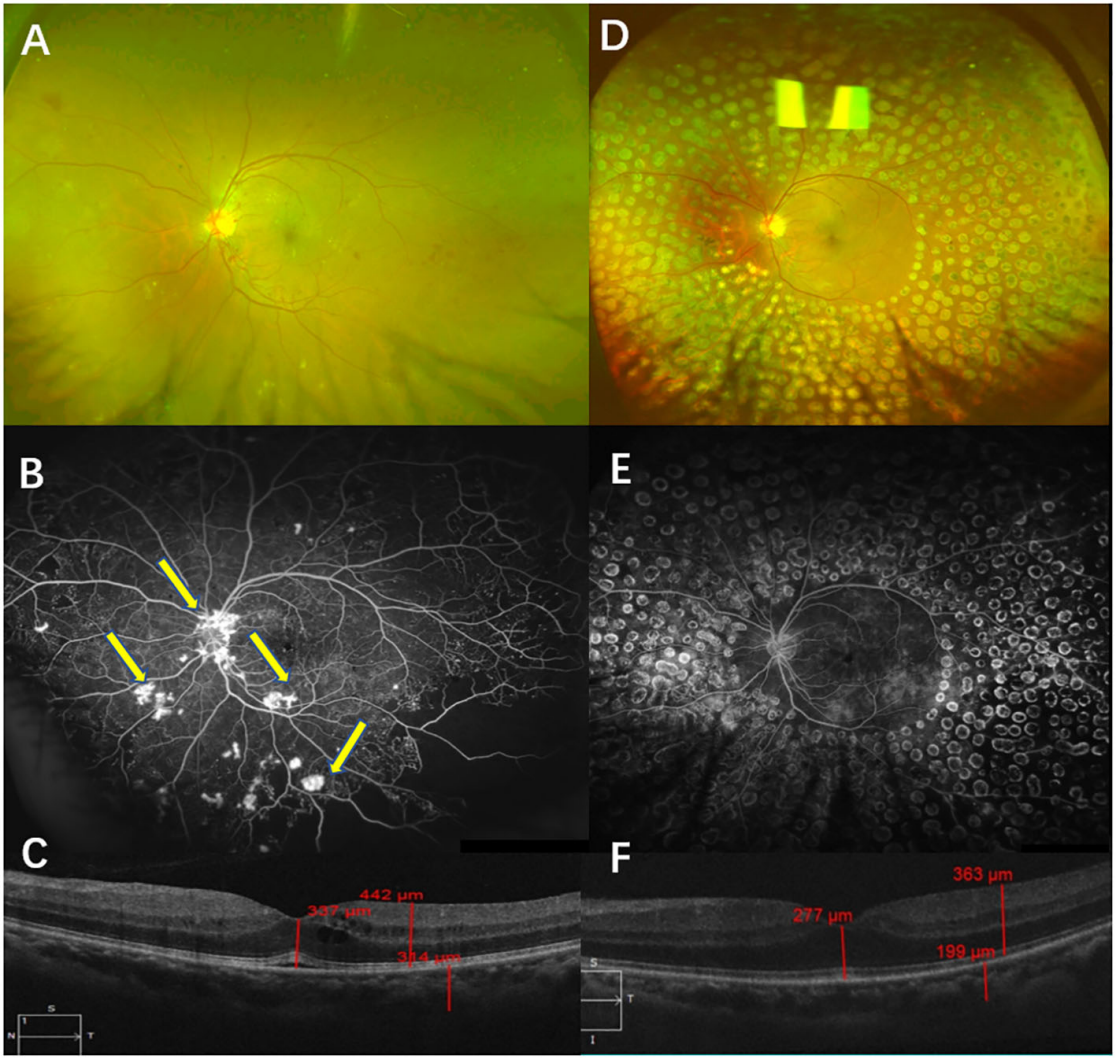
Typical case 1. Color fundus photography (CFP) **(A)** and fluorescein angiography (FA) **(B)** were performed before treatment and showed high-risk PDR in the left eye. CFP examinations showed NV, which was confirmed by FFA (**B**, arrow). OCT examinations showed macular edema **(C)**. CFP and FA examinations after a follow-up of 12 months showed that the retina had laser shots. No NV was found *via* either CFP or FA examinations **(D, E)**. OCT examinations of the left eye showed the transformation from edema to full recovery of the macula **(F)**.

A 40-year-old man presented with blurred vision in the left eye for two weeks. He had a history of diabetes for eight years. A physical examination showed that the BCVA in his left eye was 45 letters. The CFP examinations showed NV, which was confirmed by FFA. The results of SD-OCT examinations indicated macular edema, and the CMT was 337 µm. He was diagnosed with high-risk PDR in the left eye and was administered IVC (five times) and PRP. After a follow-up of 12 months, complete NV regression in the left eye was recorded. Also, his BCVA was 85 letters, and his CMT was 277 µm after 12 months (**Figure 7**).

#### Case 2

**Figure 8:**
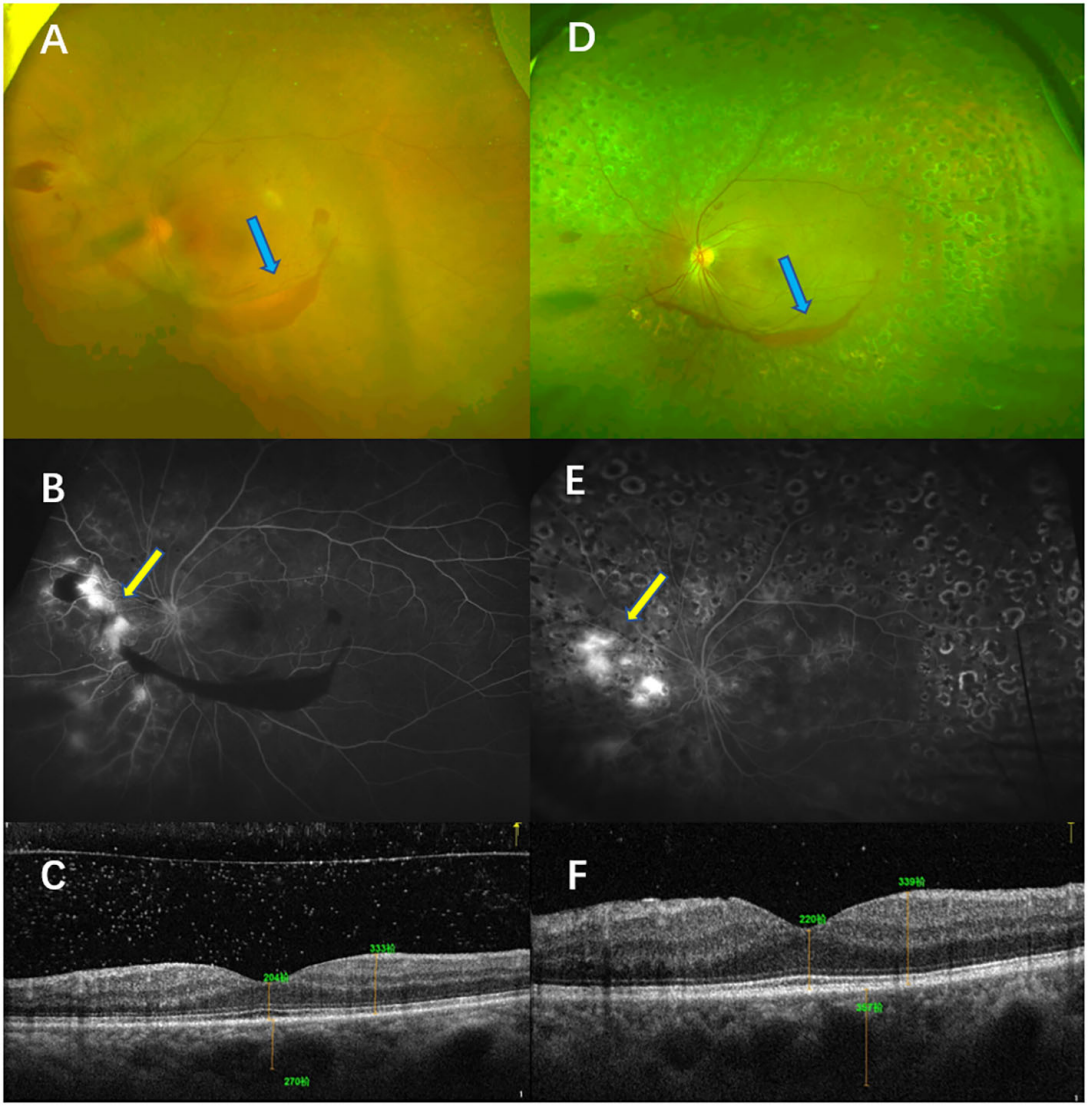
Typical case 2. The patient was diagnosed with high-risk PDR in the left eye and treated with PRP. Color fundus photography (CFP) **(A)** and fluorescein angiography (FA) **(B)** before treatment showed high-risk PDR in the left eye. The CFP examinations showed vitreous hemorrhage (blue arrow, **A**), and the FFA examinations showed NV [yellow arrow, **(B)**]. OCT showed that no macular edema was present **(C)**. CFP and FA examinations after a follow-up of 12 months showed that the retina had laser shots. The CFP examinations showed that the area of the vitreous was smaller (blue arrow, **D**), but the FA examinations showed NV leakage (yellow arrow, **E**). OCT examinations of the left eye showed that macular edema was absent **(F)**.

A 55-year-old woman presented with blurred vision in the left eye for a month. She had a history of diabetes for 15 years. Her physical examination showed that the BCVA of her left eye was 40 letters. The CFP examinations showed NV and vitreous hemorrhage, which were confirmed by FFA. The CMT was 204 µm. She showed NV even after receiving PRP monotherapy. Although rescue photocoagulation was conducted, after a 12-month follow-up, only partial NV regression was recorded in the left eye. Her BCVA was 65 letters, and her CMT was 211 µm after 12 months (**Figure 8**).

## Data availability statement

The raw data supporting the conclusions of this article will be made available by the authors, without undue reservation.

## Ethics statement

The study was approved by Peking University People’s Hospital Medical Ethics Committee. Written informed consent forms were signed by all of the patients.

## Author contributions

All authors contributed to the study conception and design. Performing the screening diagnosis and treatment of high-risk PDR (HQ, YS). Collection and assembly of data, data analysis, and interpretation (YS). Manuscript writing: All authors. All authors contributed to the article and approved the submitted version.
